# The Origin and Evolution of the 18th Century Hospital Movement

**Published:** 1914-01-31

**Authors:** 


### III. THE ORIGIN AND EVOLUTION OF THE 18th CENTURY HOSPITAL MOVEMENT.*

So far as can be ascertained, the voluntary hospital movement owed nothing at its inception to the State, the Universities, the Colleges of Physicians (Edinburgh was an exception), the Companies of Barber-Surgeons, or Apothecaries, which were the authorities in whose hands lay the power of granting licences to practise medicine and surgery. The Bishops still claimed and exercised the privilege of licensing surgeons under the Act 3 Henry VIII.; and the Archbishop of Canterbury, under a later statute, 25 Henry VIII., had the power of conferring faculties in physic with the title of M.D. Cantaur. (or Lambeth). Whether with the object of demonstrating the virility of their claims over the profession of medicine, or, as is more probable, to give encouragement to the charitable efforts of the promoters, the fact stands out that most of the early voluntary hospitals owed much of their success to the zeal and energy of the clergy. The first President of both Westminster and St. George's Hospitals was Dr. Richard Willis, Bishop of Winchester; the originator of those at Winchester and Exeter, during his tenure in turn of the Deanery of each of those Cathedrals, was Dr. Alured Clarke; the first promoter of that at Gloucester was its Bishop, Dr. Martin Benson; among the earliest subscribers to all of them may be found the names of the foremost dignitaries of the Church; and on the anniversary of each foundation a memorial sermon was usually preached by one of the Bishops.

It has been computed that from fifty to one hundred thousand French Protestant refugees had migrated to this country on the revocation of the Edict of Nantes in 1685, and upon the accession of George I. their religious zeal formed one of the stoutest bulwarks of the House of Hanover. Men who are prepared to abandon friends and country rather than forsake a religion other than that of their own nation are usually superior to their fellows in intelligence, strength, and nobility of character. Their industry and manufacturing skill made them desirable citizens; their Protestantism was a valuable asset against the Catholic (Jacobite) faction; and these together secured for them the favour of the English monarch.

One of these had in 1708 bequeathed a sum of money for the erection and maintenance of a hospital for his poor compatriots in London, and ten years later the charity was incorporated by Royal patent. By its statutes no persons were to be received except French Protestants or their descendants residing in Great Britain, and those admitted were obliged to take the oaths of allegiance, supremacy, and abjuration. Its constitution was framed upon the model of similar institutions in France, where, it is said, one had already been established in most of the provinces; but the novelty of the scheme, so far as this country was concerned, was the appeal by its promoters for the voluntary subscriptions of the public towards its support. No fees or securities of any kind were required, cases of every description being received with the single reservation of married persons, who were probably excluded with the object of preventing abuse of the charity by those who might be desirous of evading their marital obligations.

Constituted as the French Protestant Hospital was for the purpose of providing solely for the poor of a foreign population, and resembling an almshouse rather than the present conception of a hospital, it can scarcely take rank among our national institutions. But it was undoubtedly the first establishment in Great Britain for the reception of the sick poor to be supported by voluntary subscriptions, and the success it secured could scarcely fail to be in the minds of the promoters of the Westminster Infirmary who had no other example before them.

The first meeting of the trustees and managers of the charity for relieving the sick and needy was held on December 2, 1719, among those present being Mr. Henry Hoare. "After long intermission it having pleased Almighty God to stir up the hearts of some persons to revive the charitable design of relieving the sick and needy," it was decided to erect an infirmary in St. Margaret's parish to be supported by voluntary subscriptions, "there being nothing of that sort within the populous city and liberties of Westminster." The proposal ran: "Whereas great numbers of sick persons in this city languish for the want of necessaries and too often die miserably, who are not entitled to parochial relief; and whereas amongst those who do receive relief from their respective parishes, many suffer extremely and are sometimes lost, probably from want of accommodation and proper medicine in their own houses and lodgings (the closeness and unwholesomeness of which is too often one great cause of their sickness), partly by the imprudent laying out of what is allowed, and by the ignorance and ill-management of those about them, we, whose names are underwritten, in obedience to the rules of our holy religion, desiring, so far as in us lies, to find some remedy for this great misery of our poor neighbours, do subscribe the following sums of money to be paid by us yearly (during pleasure) by quarterly payments for the procuring, furnishing, and defraying the necessary expenses of an infirmary or place of entertainment for such poor sick persons inhabiting the parish of St. Margaret, Westminster, or others who shall be recommended by any of the subscribers or benefactors, with the approbation and consent of the major part of the trustees present (it is resolved that all subscribers shall be trustees of this charity); who are likewise empowered to allow suitable relief to such sick persons, approved in the manner above mentioned, as are incapable of being removed from their respective abodes." A comparison of the proposal of the Charitable Society with the above shows that they were the same in principle, with the addition in the later scheme of the provision of a place of entertainment for the sick poor, free of all expense.

The stay of each patient in the infirmary was limited in the first instance to one month, which period was extended as occasion required. It was to be less of an almshouse and more of a hospital than any of its predecessors, and it was probably with this idea that the trustees were empowered to allow suitable relief to sick persons incapable of removal to the infirmary.

The only patients specifically excluded were incurables, lunatics, lying-in women, and those suffering from venereal and infectious diseases, under which latter heading were comprised smallpox, itch, and scald-head, these alone being mentioned by name.

Security for burial or removal was required from all cases but those of sudden accident, which were admitted without recommendation; and, contrary to the custom at

* Previous articles appeared on December 13, 1913, and January 17, 1914.

the chartered hospitals, no fees or charges of any description were demanded.

Contributions were paid quarterly (probably in order to keep pace with the current expenditure), and each subscriber was entitled to have one in- and one out-patient on the books at the same time. In addition to the out-patients thus recommended, the physicians and surgeons, on certain days at stated hours, gave advice gratis to all such poor diseased persons as thought fit to attend. These received the prescription of a physician or the assistance of a surgeon, but apparently were not supplied with medicine, which they were left to obtain at their own expense. As has been already explained, this practice was probably adopted in imitation of the dispensary scheme, but was not continued by most of the later hospitals.

The voluntary principle was further extended by the acceptance of the offer of the gratuitous services of certain eminent physicians and surgeons, which honorary appointments were innovations upon the standing custom at the chartered hospitals, where each member of the staff was a salaried officer, and as such had no voice in the general management.

